# Prosthetic Rehabilitation and Its Effect on Head Posture

**DOI:** 10.7759/cureus.24630

**Published:** 2022-04-30

**Authors:** Cimmy Augustine, Anil K Gujjari, Nayana Paul, Sowmya Neelan, Raghavendra Swamy

**Affiliations:** 1 Prosthodontics and Crown & Bridge, Mahe Institute of Dental Sciences, Mahe, IND; 2 Prosthodontics and Crown & Bridge, Jagadguru Sri Shivarathreeshwara Dental College, Mysuru, IND; 3 Prosthodontics and Crown & Bridge, Buddha Institute of Dental Sciences and Hospital, Patna, IND

**Keywords:** edentulousness, natural head position, complete dentures, postural stability, prosthetic rehabilitation, head posture, edentulism

## Abstract

Background: Edentulism is a debilitating and irreversible condition. It is often accompanied by compromises in the surrounding joint’s range of motion and changes in the posture of the head. The natural head position is maintained by a balanced tension between cranio-cervical bones, myofacial structures and the dental occlusion. Loss of teeth may cause changes in the head posture that may disturb the patency of the spinal cord and lead to the loss of postural balance. Therefore, this study aimed at evaluating the head posture in the edentulous subjects before and after prosthetic rehabilitation.

Methods: A total of 16 completely edentulous subjects were selected for the study. Removable complete denture prosthesis was fabricated for all the subjects. Lateral photographs were taken at different time intervals i.e., pre-rehabilitation, 30 minutes, 2 days and 30 days post-rehabilitation. The cranio-vertical angle obtained was digitally calculated using Kinovea software and the results obtained were statistically analysed.

Results: The paired-sample t-test and repeated measures analysis of variance showed an increase in the cranio-vertical angulation of edentulous subjects after rehabilitation, indicating a mild extension of the head.

Conclusion: The insertion of prosthesis leads to a mild extension of the head. Hence, rehabilitation with a removable prosthesis has a positive effect on the head posture and could therefore aid in maintaining a stable head posture.

## Introduction

Population ageing is a global phenomenon. According to the United Nations (UN), the percentage of elderly people, classified as those above 60 years of age, is expected to go up in India from 8% in 2015 to 19% in 2050, as highlighted in a report released by the UN Population Fund India [1]. One of the common consequences of ageing is edentulousness. Edentulism is a debilitating and an irreversible condition and is described as the “ultimate marker of disease burden for oral health” [2]. Although the prevalence of complete tooth loss has declined over the last decade, edentulism still remains a major disease worldwide, especially among older adults. Edentulousness is associated with physiological bone resorption and various muscular, soft tissue changes [3]. It is often accompanied by compromises in the surrounding joint’s range of motion and changes in the posture of the head.

The natural head position is maintained by a balanced tension between cranio-cervical bones, myofacial structures and the dental occlusion [4]. When the dental occlusal relationship is lost, it may lead to changes in the orientation of the skull on the neck. A change in the head posture results in a change in neuromuscular articulations of the skull and may disturb the patency of the vertebrae. It also alters the body’s centre of gravity and may lead to loss of postural balance.

Previous reports have suggested that rehabilitation with complete dentures not only helps to improve mastication, aesthetics and phonetics, but also alters the head posture and body balance. Manni et al. found that the presence of dentures influenced the stability of mild-to-moderate dementia participants [5]. Watanabe and Fujinami et al. found that wearing complete denture increases the gait velocity and provided better body balance [6-7]. Okubo et al. found that complete dentures produced an effect on the stability of patients in static and dynamic conditions [8].

A previous unpublished study (Augustine C, Gujjari A, Dhakshaini M, Kuriakose E. Evaluation of head posture in dentate and edentulous) has evaluated the changes in head position among dentate and edentulous participants. However, information as to the effect of complete dentures on the natural position of head is limited. Therefore, the present study aimed at evaluating the head position and the effect of prosthetic rehabilitation on the posture of the head in edentulous participants.

## Materials and methods

The study was carried out at the Department of Prosthodontics and Crown & Bridge, Jagadguru Sri Shivarathreeshwara (JSS) Dental College and Hospital, Mysuru, India. A total of 16 adults aged 40-65 years with good neuromuscular control and without any gross facial asymmetry who were completely edentulous for at least three months and with no previous denture experience were included in the study. Those with Class 2 or 3 skeletal relationships, with any kind of neuromuscular disorders or cranio-cervical diseases, nasal allergies, chronic respiratory infections or head and neck malignancies were excluded from the study. Written informed consent was obtained from those who agreed to participate voluntarily. Ethical clearance was obtained from the Institutional Ethical committee of the JSS Academy of Higher Education and Research.

Removable prosthesis was fabricated for the participants by following the conventional methods for complete denture fabrication. Post-denture fabrication, the fit of the dentures was analysed intra-orally. Lateral photographs were taken at different time intervals, i.e., pre-rehabilitation (Group 1), 30 minutes post-rehabilitation (Group 2A), 2 days post-rehabilitation (Group 2B), and 30 days post-rehabilitation (Group 2C).

Positioning for photographs

**Figure 1:**
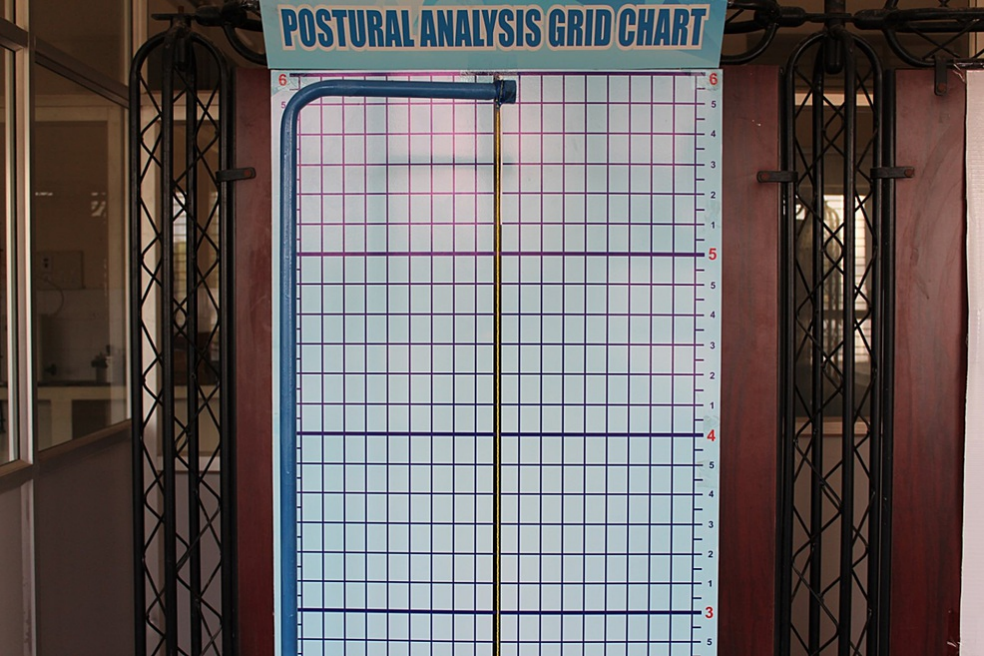
Posture analysis grid chart and plumb line

**Figure 2:**
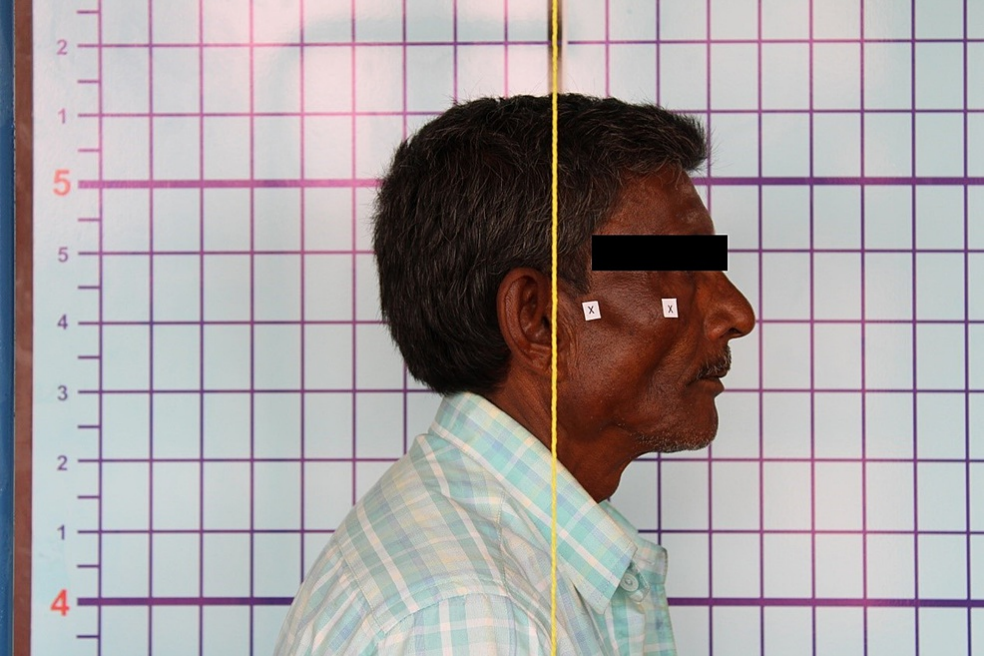
Digital photograph

A wall-mounted posture analysis grid chart with a darkened central vertical line was used to correctly position the patient in accordance with the plumb line (Figure 1). A plumb line was suspended from an L-shaped metal rod. It was used for determining the vertical reference line. A digital single-lens reflex camera (Canon D1300) was positioned at a distance of five feet from the grid chart. Two cross-markings were placed on the patient’s face. The first cross-marking was placed on the zygomatic prominence and the second cross-marking was placed 2 cm anterior to the upper border of the tragus [9]. The patient was positioned to stand in front of the grid with the left shoulder towards the grid (Figure 2). Instructions were given to look in a direction perpendicular to the grid and camera setup. Before taking the photographs, the participant was asked to tilt their head forward and backward with a decreasing amplitude until reaching a position of comfortable head balance, thus, establishing a self-balanced head position [10].

**Figure 3:**
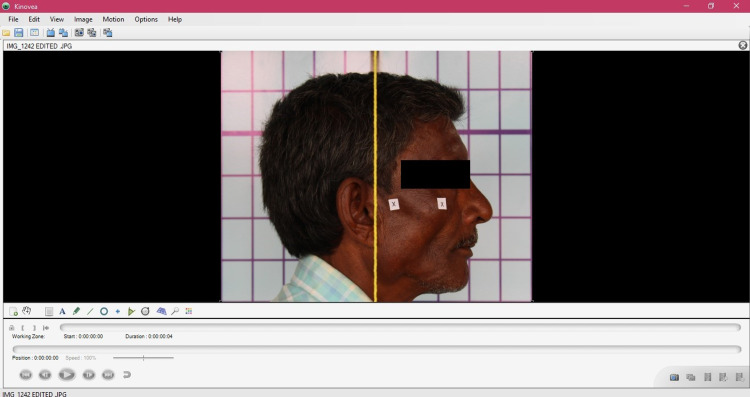
Software analysis of the photograph

**Figure 4:**
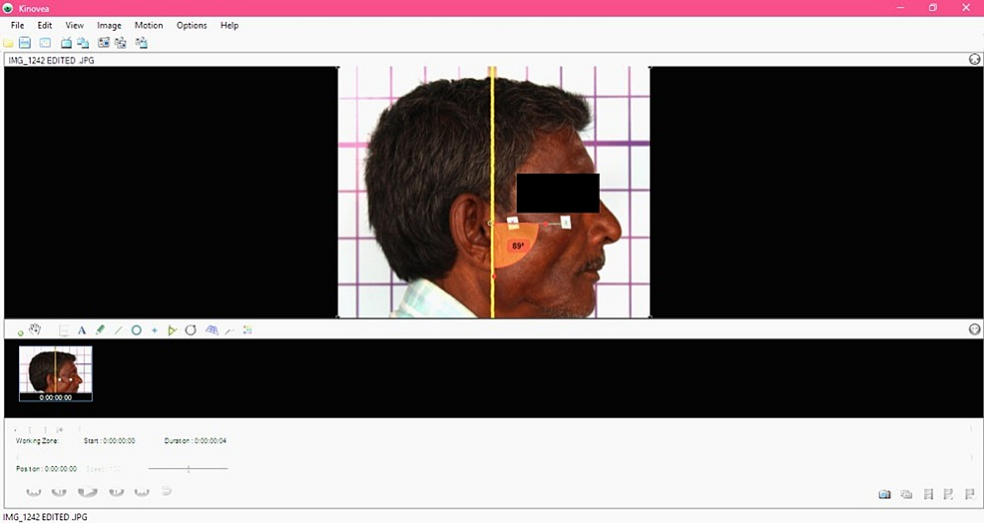
Determination of the cranio-vertical angle using the software

Once the photographs were obtained, the images were digitized and analysed by using an image-analysing software Kinovea (version 0.8.15, free and open source; Figure 3). Using the software application, a straight line was drawn digitally by joining both the cross-markings and this line was extended till it bisected the vertically hanging plumb line in the image. The straight line acted as the horizontal reference line and the plumb line acted as the vertical reference line. The angle formed between the reference lines was digitally measured to determine the cranio-vertical angle and the cranio-vertical angle obtained was tabulated for each group (Figure 4). The difference in the head position at different intervals of time was calculated, mean was obtained and the data was statistically analysed.

## Results

Statistical analysis

Statistical analysis was performed using SPSS Statistics, version 22 for Windows (IBM Corp., Armonk, NY), and the results were tabulated (Table 1). Descriptive statistics such as means and standard deviations of the cranio-vertical angles were calculated for all the groups. A paired-sample t-test was applied to measure the cranio-vertical angle at different time intervals. Repeated measures analysis of variance (ANOVA) was also carried out to measure the cranio-vertical angle at four different time intervals, i.e., pre-insertion, 30 minutes after insertion, 2 days after insertion and 30 days after insertion.

**Table 1:** Repeated measures ANOVA and post hoc comparison of edentulous subjects at different time intervals pre- and post-rehabilitation The results were found to be statistically significant between the groups (p≤0.05). The results were even significant when a comparison was made between all possible pairs (p≤0.05) except between 2 days post-rehabilitation vs 30 days post-rehabilitation where the result was found to be non-significant (p>0.05). ANOVA, analysis of variance

Group	Time intervals				Statistical inference
	Pre-rehabilitation	30 mins post-rehabilitation	2 days post-rehabilitation	30 days post-rehabilitation	
	Mean ± SD	Mean ± SD	Mean ± SD	Mean ± SD	
	N = 16	N = 16	N = 16	N = 16	
Edentulous participants	85.69 ± 2.36	89.37 ± 2.99	88.50 ± 3.08	88.12 ± 3.16	F value: 32.01
					df: 3
					p value: 0.00
Post hoc comparison	Pre-rehabilitation vs 30 minutes post-rehabilitation = 0.00				
	Pre-rehabilitation vs 2 days post-rehabilitation = 0.00				
	Pre-rehabilitation vs 30 days post-rehabilitation = 0.02				
	30 minutes post-rehabilitation vs 2 days post-rehabilitation = 0.01				
	30 minutes post-rehabilitation vs 30 days post-rehabilitation = 0.02				
	2 days post-rehabilitation vs 30 days post-rehabilitation = 0.98				

Table 2 shows paired statistical analysis of mean cranio-vertical angles among completely edentulous subjects at different paired time intervals. The paired-samples t-test showed that the mean cranio-vertical angle recorded in Group 2A subjects, 30 minutes post-rehabilitation with complete dentures, was 3.68° higher than the pre-rehabilitation value. The value obtained was statistically significant (p<0.05). The mean cranio-vertical angle recorded 2 days post-rehabilitation was 2.81° higher than the pre-rehabilitation value and it was statistically significant (p<0.05). The mean cranio-vertical angle recorded 30 days post-rehabilitation was 2.43° higher than the pre-rehabilitation value and the result was statistically significant (p<0.05). The mean cranio-vertical angle recorded 30 minutes post-rehabilitation was 0.87° higher than that recorded 2 days post-rehabilitation. The value obtained was statistically significant (p<0.05). The cranio-vertical angle 30 minutes post-rehabilitation was 1.25° higher than that recorded 30 days post-rehabilitation and the value obtained was statistically significant (p<0.05). The cranio-vertical angle 2 days post-rehabilitation was 0.37° higher than that recorded 30 days after rehabilitation and it was statistically not significant (p>0.05). The mean cranio-vertical angle recorded in Group 2 subjects after rehabilitation with prosthesis was higher than that recorded before rehabilitation. The mean cranio-vertical angle recorded in Group 2 subjects was the highest at a time interval of 30 minutes after rehabilitation.

**Table 2:** Comparison of cranio-vertical angles between different possible edentulous groups *Paired t-test Pair 1 refers to pre-rehabilitation (Group 1) and 30 minutes after rehabilitation with complete dentures (Group 2A); pair 2 refers to pre-rehabilitation (Group 1) and 2 days after rehabilitation with complete dentures (Group 2B); pair 3 refers to pre-rehabilitation (Group 1) and 30 days after rehabilitation with complete dentures (Group 2C); pair 4 refers to 30 minutes after (Group 2A) and 2 days after rehabilitation with complete dentures (Group 2B); pair 5 refers to 30 minutes (Group 2A) and 30 days after rehabilitation with complete dentures (Group 2C); pair 6 refers to 2 days (Group 2B) and 30 days after rehabilitation with complete dentures (Group 2C).

Group		Mean ± SD	Statistical inference*		
Pair 1	Group 1	85.69 ± 2.36	t value: 7.96	df: 15	p value: 0.00
	Group 2A	89.38 ± 2.99			
Pair 2	Group 1	85.69 ± 2.36	t value: 5.51	df: 15	p value: 0.00
	Group 2B	88.50 ± 3.08			
Pair 3	Group 1	85.69 ± 2.36	t value: 4.72	df: 15	p value 0.00
	Group 2C	88.13 ± 3.16			
Pair 4	Group 2A	89.38 ± 2.99	t value: 3.66	df: 15	p value: 0.02
	Group 2B	88.50 ± 3.08			
Pair 5	Group 2A	89.38 ± 2.99	t value: 4.70	df: 15	p value: 0.00
	Group 2C	88.13 ± 3.16			
Pair 6	Group 2B	88.50 ± 3.08	t value: 1.46	df: 15	p value: 0.16
	Group 2C	88.13 ± 3.16			

**Figure 5:**
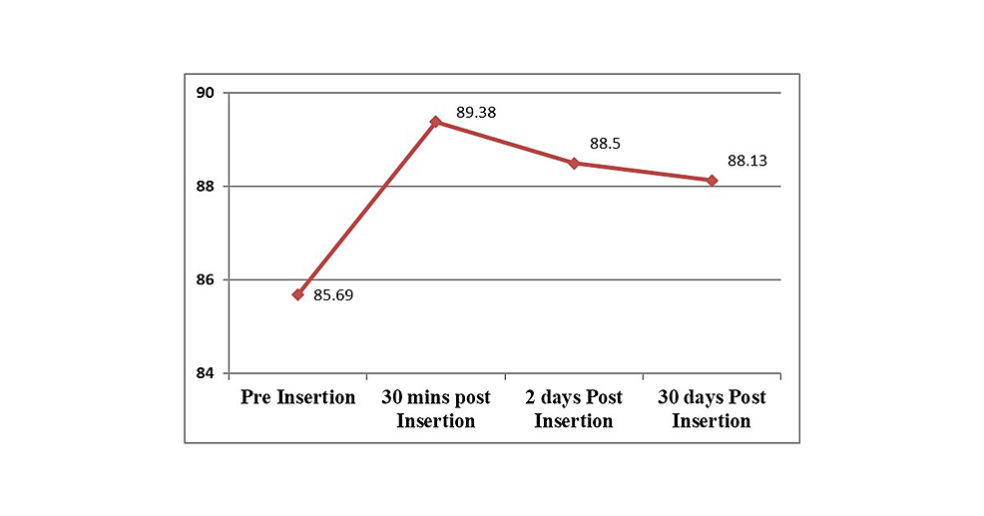
Change in the cranio-vertical angulation among edentulous study participants’ pre- and post-rehabilitation at different time intervals

Changes in the cranio-vertical angle of completely edentulous subjects pre- and post-rehabilitation at different time intervals, i.e., 30 minutes, 2 days and 30 days are shown in Figure 5. For Group 1 (pre-rehabilitated completely edentulous participants), it was observed that the mean value of the cranio-vertical angle obtained was 85.69°. For Group 2A (30 minutes post-rehabilitation with complete dentures), a mean cranio-vertical angle of 89.38° was recorded. The observed values when compared to the edentulous participants’ pre-rehabilitation values had significantly increased by 3.7°. This suggests the extension of the head. For Group 2B (2 days post-rehabilitation with the complete denture), the mean cranio-vertical angle value obtained was 88.5°. The observed values indicated that when compared to the edentulous subjects’ pre-rehabilitation values, the cranio-vertical angle values had increased by 2.8°, suggesting the extension of the head. For Group 2C (30 days post-rehabilitation with complete dentures), the mean cranio-vertical angle value obtained was 88.13°. The observed values indicated that when compared to the edentulous subjects’ pre-rehabilitation values, the cranio-vertical angle values had increased by 2.4°, suggesting the extension of the head. However, when compared to the baseline values, the cranio-vertical angle was higher even after 30 days.

## Discussion

The cervical muscles maintaining head balance and the muscles of the stomatognathic system could be regarded as a coordinated system in which intervention at any level will bring about a change in the whole system [11]. It may be postulated that the loss of teeth and alveolar structures would lead to changes that could affect the head position.

Conte stated that the function of the head, neck, and jaws is closely interrelated forming a combined system, i.e., cranio-cervical-mandibular functional system. The collapse of the skull could be because of either physical causes (force of gravity, muscular force) or mechanical causes (lack of dental height). Thus, if the dental arches do not have adequate dental height, the skull finds no support and tends to sink downwards and forwards, pulling the cervical spine along with it. This alters the centre of gravity of the body and the body balance. Therefore, to bring the skull back to its ideal position, it is necessary to substitute the missing dental height [12].

This study was planned with the purpose of evaluating the natural head position in edentulous patients and compare the changes in the cranio-vertical angle after the insertion of complete dentures. The results obtained in our study indicated an increase in the cranio-vertical angle, i.e., a mild extension of the head even 30 days after the insertion of prosthesis. When compared to previous studies, the results obtained in this study were different. The reasons for the differences between the results are not clear, but several factors might play a role. It may be a result of the difference in the methods used to establish natural head position. Differences in tongue size, tongue form, lip and tongue pressure, larger and thicker lingual flanges or the differences in cranio-facial morphology from one individual to another could also be the variation factors [13-16]. Salonen et al. found that the patients who experienced the greatest reduction in their free-way space, i.e., vertical dimension of occlusion increased, were seen to have raised their heads (extension) more than average [17].

Studies have proposed that ageing and loss of teeth lead to a reduction in the muscle mass, which could affect the sway at the centre of gravity of the body resulting in an increased risk of fall [18,19]. Therefore, in order to stabilise the posture, a compensatory tilting of the head and bending of the cervical column, i.e., flexion of the head, might be seen. It is already known that flexion increases the compressive forces applied to the nerve root whereas extension decreases the force [20]. Hence, this adaptive postural change could lead to discomfort or pain because of nerve compression [21].

The present study proposes that with the insertion of complete dentures, the lost vertical dimension is restored and thus the position of the jaws in the skull and the neuromuscular stability can be maintained. This could also compensate for the adaptive postural change caused due to the loss of dental height, thereby altering the centre of gravity of the body and improving the postural stability and body balance. It could also aid in relieving the discomfort caused due to nerve compression. Thus, dental intervention may improve the life activity of the elderly.

## Conclusions

Based on the findings of our study, it can be said that the insertion of prosthesis leads to a mild extension of the head. Therefore, we postulate a hypothesis that complete dentures may be an effective aid in restoring the head position, maintaining the postural balance and possibly alleviating neck pain. Although this study has achieved its objectives, there were some limitations. This study included a small sample size of the population. Also, the subjects selected for the study were aged 40-65 years, but in older age groups, the pattern of changes in the cranio-vertical angle may be different. Further research, with a large sample size, could help in confirming the effects of prosthetic rehabilitation in the maintenance of a stable head position, which could improve the quality of life for elderly people.
